# Geriatric assessment with management for older patients with cancer receiving radiotherapy: a cluster-randomised controlled pilot study

**DOI:** 10.1186/s12916-024-03446-4

**Published:** 2024-06-10

**Authors:** Marit Slaaen, Inga Marie Røyset, Ingvild Saltvedt, Bjørn Henning Grønberg, Vidar Halsteinli, Øystein Døhl, Corinna Vossius, Øyvind Kirkevold, Sverre Bergh, Siri Rostoft, Line Oldervoll, Asta Bye, Line Melby, Tove Røsstad, Guro Falk Eriksen, May Ingvild Volungholen Sollid, Darryl Rolfson, Jūratė Šaltytė Benth

**Affiliations:** 1 The Research Centre for Age-Related Functional Decline and Disease, Innlandet Hospital Trust, Box 68, 2312 Ottestad, Norway; 2 Institute of Clinical Medicine, Faculty of Medicine, University of Oslo, Pb 1171 Blindern, Oslo, 0318 Norway; 3 Department of Neuromedicine and Movement Science, Faculty of Medicine and Health Science, Norwegian University of Science and Technology (NTNU), N-7491 Trondheim, Norway; 4 Department of Geriatric Medicine, Clinic of Medicine, St. Olavs Hospital, Trondheim University Hospital, Box 3250 Torgarden , Trondheim, NO-7006 Norway; 5 Department of Oncology, St. Olav Hospital, St. Olavs Hospital, Trondheim University Hospital, Box 3250 Torgarden , Trondheim, NO-7006 Norway; 6 Department of Clinical and Molecular Medicine, Norwegian University of Science and Technology (NTNU), Trondheim, Norway; 7 Regional Center for Health Care Improvement, St. Olavs Hospital, Trondheim University Hospital, Box 3250 Torgarden , Trondheim, NO-7006 Norway; 8 Trondheim Municipality, Trondheim Kommune, Postboks , Trondheim, Norway; 9 Centre for Age-Related Medicine, Stavanger University Hospital, Stavanger, Norway; 10 Norwegian National Centre for Ageing and Health, Vestfold Hospital Trust, Postboks , Tønsberg, 2136, 3103 Norway; 11 Department of Health Sciences in Gjøvik, NTNU, Box 191, N-2802 Gjøvik, Norway; 12 Department of Geriatric Medicine, Oslo University Hospital, Pb , Nydalen, Norway; 13 Center for Crisis Psychology, Faculty of Psychology, University of Bergen, 7807, 5020 Bergen, PB Norway; 14 Department of Public Health and Nursing, NTNU, 8905, 7491 Trondheim, PB Norway; 15 Oslo Metropolitan University (Oslomet), Postboks 4, St. Olavs Plass, 0130 Oslo, Norway; 16 European Palliative Care Research Centre (PRC), Institute of Clinical Medicine, Faculty of Medicine, Department of Oncology, University of Oslo, and, Oslo University Hospital, Nydalen, Norway; 17 Department of Internal Medicine, Hamar Hospital, Innlandet Hospital Trust, Skolegata 32, 2318 Hamar, Norway; 18 Division of Geriatric Medicine, Clinical Sciences Building, University of Alberta, 1-19811350 83 Ave, Edmonton, AB T6G 2P4 Canada; 19 Institute of Clinical Medicine, University of Oslo, Campus Ahus, P.O.Box 1171, 0318 Blindern, Norway; 20 Health Services Research Unit, Akershus University Hospital, P.O.Box 1000, 1478 Lørenskog, Norway

**Keywords:** Geriatric assessment with management, Older patients, Frailty, Cancer, Radiotherapy, Randomised controlled trial

## Abstract

**Background:**

Geriatric assessment and management (GAM) improve outcomes in older patients with cancer treated with surgery or chemotherapy. It is unclear whether GAM may provide better function and quality of life (QoL), or be cost-effective, in a radiotherapy (RT) setting.

**Methods:**

In this Norwegian cluster-randomised controlled pilot study, we assessed the impact of a GAM intervention involving specialist and primary health services. It was initiated in-hospital at the start of RT by assessing somatic and mental health, function, and social situation, followed by individually adapted management plans and systematic follow-up in the municipalities until 8 weeks after the end of RT, managed by municipal nurses as patients’ care coordinators. Thirty-two municipal/city districts were 1:1 randomised to intervention or conventional care. Patients with cancer ≥ 65 years, referred for RT, were enrolled irrespective of cancer type, treatment intent, and frailty status, and followed the allocation of their residential district. The primary outcome was physical function measured by the European Organisation for Research and Treatment of Cancer Quality of Life Questionnaire-C30 (QLQ-C30). Secondary outcomes were overall quality of life (QoL), physical performance, use and costs of health services. Analyses followed the intention-to-treat principle. Study registration at ClinicalTrials.gov ID NCT03881137.

**Results:**

We included 178 patients, 89 in each group with comparable age (mean 74.1), sex (female 38.2%), and Edmonton Frail Scale scores (mean 3.4 [scale 0–17], scores 0–3 [fit] in 57%). More intervention patients received curative RT (76.4 vs 61.8%), had higher irradiation doses (mean 54.1 vs 45.5 Gy), and longer lasting RT (mean 4.4 vs 3.6 weeks). The primary outcome was completed by 91% (intervention) vs 88% (control) of patients. No significant differences between groups on predefined outcomes were observed. GAM costs represented 3% of health service costs for the intervention group during the study period.

**Conclusions:**

In this heterogeneous cohort of older patients receiving RT, the majority was fit. We found no impact of the intervention on patient-centred outcomes or the cost of health services. Targeting a more homogeneous group of only pre-frail and frail patients is strongly recommended in future studies needed to clarify the role and organisation of GAM in RT settings.

**Supplementary Information:**

The online version contains supplementary material available at 10.1186/s12916-024-03446-4.

## Background

The global increase in the number and proportion of older adults [1, 2] challenges our health care services [1], and adjustment of treatment and care to varying health statuses is paramount. Addressing this, comprehensive geriatric assessment (CGA) plays a crucial role in health care delivery as a “multidimensional, interdisciplinary, diagnostic process to identify care needs, plan care, and improve outcomes of frail older people” [3, 4].

In geriatric medicine, CGA has proven successful in reducing mortality, functional deterioration, and the need for institutional care [3, 5, 6]. Adapted to cancer care, CGA is often referred to as geriatric assessment (GA) with management (GAM), i.e. management of impairments identified by a systematic assessment of comorbidities, medications, nutritional status, physical and cognitive function, depressive symptoms, and social support [7, 8, 9]. Several randomised controlled trials (RCT), predominantly addressing older patients receiving cancer surgery or chemotherapy, have shown that GAM interventions may facilitate treatment completion, and reduce adverse events and the need for hospital services [10, 11]. Benefits related to quality of life (QoL) and physical performance are more poorly documented [10, 11]. Few trials have included such patient-centred outcomes [12, 13, 14, 15, 16], although recommended in cancer trials addressing older patients in particular [17, 18]. Cost effects are also scarcely investigated [11, 19], and evidence of any impact of GAM in radiotherapy (RT) settings is lacking.

RT is a main treatment modality in cancer, estimated to be needed by 45–60% of patients during the course of their disease [20]. It is generally considered more tolerable than surgery and chemotherapy, but may still have serious side effects [21]. Toxicity, impaired QoL, and physical deterioration are serious concerns, particularly in older patients [22], and frequent co-existing, age-related problems have been shown to affect survival in this patient group [23]. Correspondingly, a gradual decline in QoL and physical function has been demonstrated with an increasing number of geriatric impairments [24]. GA in older patients receiving RT has therefore been advocated to predict outcomes and enable targeted interventions [22].

Against this backdrop, we developed a GAM intervention aiming to improve QoL and function for patients with cancer ≥ 65 years receiving RT with palliative or curative intent [25]. The intervention involved both specialist and primary care services and included 1) an in-hospital GA at the start of RT, followed by an individually adapted management plan, (2 a systematic follow-up by municipal nurses working in cancer care (cancer contact nurses), and 3) coordination of services and collaboration across sectors (specialist and primary care) with cancer contact nurses as main actors. A pilot study was found necessary to evaluate several aspects, including patient selection, feasibility, and potential effect on pre-defined outcomes, before performing a full-scale RCT. Thus, we compared our intervention to conventional care in a controlled pilot study [25]. Since primary care professionals (cancer contact nurses) had a central role in the intervention, we randomised primary health care districts (clusters) to avoid contamination between treatment groups. The detailed objectives addressed in the present paper, pertaining to individual patients, were to answer the following questions:

- Did the intervention affect patient-centred outcomes, i.e. short- and long- term physical function, global QoL, and symptom burden, and what would be the potential effect size?

- Did the intervention influence the use and costs of health care services, and if so, to what extent?

The other main objective, to evaluate feasibility, will be fully addressed in a pending paper. A brief evaluation of inclusion criteria and adherence to the intervention programme is included in the present one.

## Methods

A detailed description of the study design is previously published [25]. The protocol, sample size estimates, and statistical analysis plan can also be found on ClinicalTrials.gov (ID NCT03881137).

### Study context

The study was performed within the Norwegian public health care, a primarily tax-financed, universal health coverage including both specialist- and primary care services (see Additional file 1: Table S1). Specialist services, covering in- and outpatient hospital services, are commanded by the government. Primary health care is managed by the municipalities and includes general practitioners (GPs), out-of-hours medical service, home care (nursing, basic assistance), nursing homes, and municipal rehabilitation services. All residents are entitled to a GP who provides general medical services including referrals to specialist services when needed. Home care and nursing homes are needs-based. Eligibility is determined based on national legislation and the municipality’s criteria, administered by health and welfare offices in the municipalities. Most municipalities employ one or two nurses designated to work with patients with cancer, who are usually referred to as cancer contact nurses. Their positions vary from part-time to full-time, and conventionally, their involvement in individual patients’ care (e.g. information, supportive and palliative care, care coordination) depends on ad hoc referrals from other professionals or contact taken by the patients themselves.

### Study design

The cluster-randomised pilot study was designed to test a multicomponent, individually targeted GAM intervention for older patients with cancer receiving RT. The intervention was developed in close collaboration with user representatives, and a reference group consisting of hospital and primary care professionals. As GAM in cancer care has no universally accepted, detailed recipe [10, 26], we based our intervention on recommendations from the international field of geriatric oncology [7, 27], experience and evidence from previous studies on GA and GAM by members of our study group [6, 28, 29, 30, 31], and adapted it to the availability and organisation of the local health service (Additional file 1: Table S1).

Patients were recruited at two RT centres, a local hospital in eastern Norway (Centre 1), serving mostly rural municipalities, and a university hospital in central Norway (Centre 2), located in a larger city (Additional file 1: Table S1). The recruitment took place from May 2019 to April 2021 with an interruption from March to September 2020 due to the COVID-19 pandemic. For each patient, a cancer contact nurse from the patient’s municipality/city district was assigned a central role in the intervention program, Thus, to ensure that the nurses who were such involved, did not treat patients in the control group, randomisation on the level of primary health care units (municipal units/city districts) was mandatory. Since both acute and long-term side effects of RT may influence patients’ function and QoL, and consequently the use and cost of health services, the patients were followed with study-specific assessments up to 1 year after the end of RT.

### Study participants

**Fig. 1:**
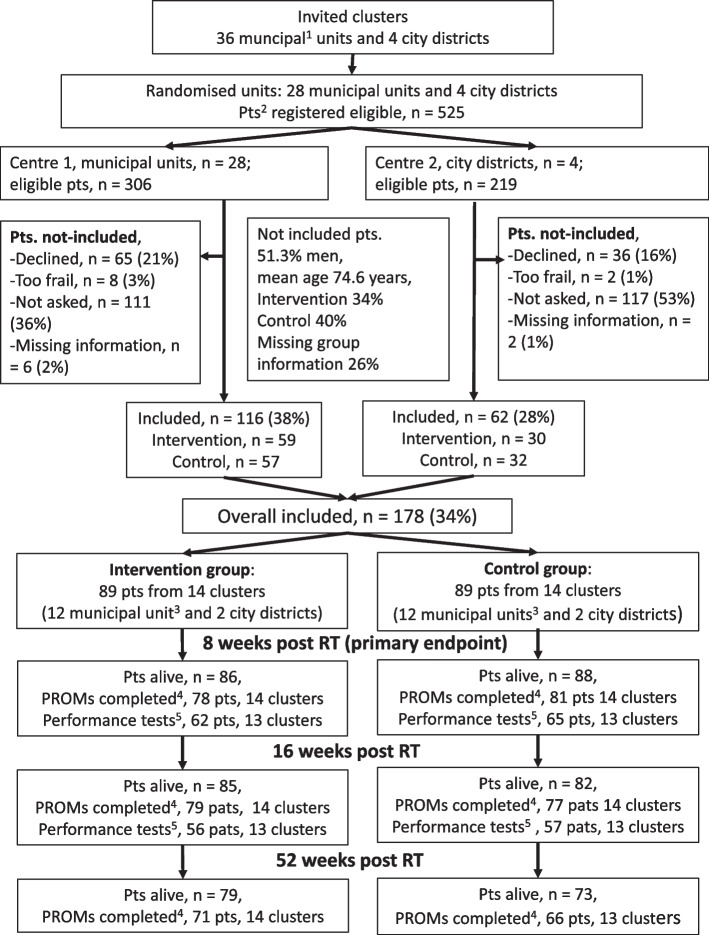
^1^Four municipalities were joined two and two into two randomised units due to sharing a common cancer nurse,^ 2^Pts, patients; ^3^No patient were included from two control and two intervention municipal units; ^4^PROMS, patient-reported outcomes, here referring to the QLQ-C30 questionnaire; ^5^Performance test, here referring to the Short Physical Performance Battery

To be eligible for study participation, the primary health care districts had to be located in the catchment area of the study centres. At Centre 1, we invited 36 municipalities that previously had been involved in research on older patients with cancer [31], and 28 consented to participate (< 4500 inhabitants [*n* = 10] up to about 35,000 inhabitants [*n* = 1]) (Fig. 1). At Centre 2, we invited four primary care city districts (34,000 to 50,000 inhabitants) to ensure representation of larger urban areas in our study sample.

Patients’ inclusion criteria were residing in one of the randomised municipal/city districts, age ≥ 65 years, referral for curative or palliative RT with a confirmed cancer diagnosis, fluency in Norwegian, and ability to answer self-report questionnaires. Exclusion criteria were referral for only one RT fraction, and/or life expectancy < 3 months. All patients provided written informed consent.

### Randomisation, recruitment, and blinding

Before the study started, the overall 32 primary health care units were stratified by the project management into five blocks according to the number of inhabitants [25], and thereafter, 1:1 randomly assigned to either intervention or control within each block by a computer-generated algorithm, 16 in each group. Eligible patients were identified by referral to the RT unit. They were consecutively recruited and approached by a study nurse (cancer nurse) at Centre 1 and a PhD student (geriatrician) at Centre 2 on the first consultation (when CT scans for RT planning were performed). Eligibility was confirmed by the patients’ oncologist, and oral and written information about the study was given. Consenting patients were informed about their allocation in accordance with their residential municipality/city district. Patients allocated to the control group received conventional care (see Additional file 1, Table S1). The other group entered the intervention program. For either group, study participation did not impact their cancer treatment. There was no blinding of patients or health professionals, except for the physiotherapists performing physical performance tests (secondary outcomes), 8 and 16 weeks after RT.

### The geriatric assessment with management (GAM) intervention

The intervention was developed to target patients receiving RT, which is mostly provided as daily outpatient treatment over a few days to several weeks. Side effects and burden of treatment are known to increase towards the end and are often most pronounced the first weeks afterwards. The intervention was therefore scheduled from the start of RT until 8 weeks after the end [25], when acute side effects would have receded for most patients. It was performed in a collaboration between hospital- and municipality-based health services and individually adapted and had three parts.

**Fig. 2:**
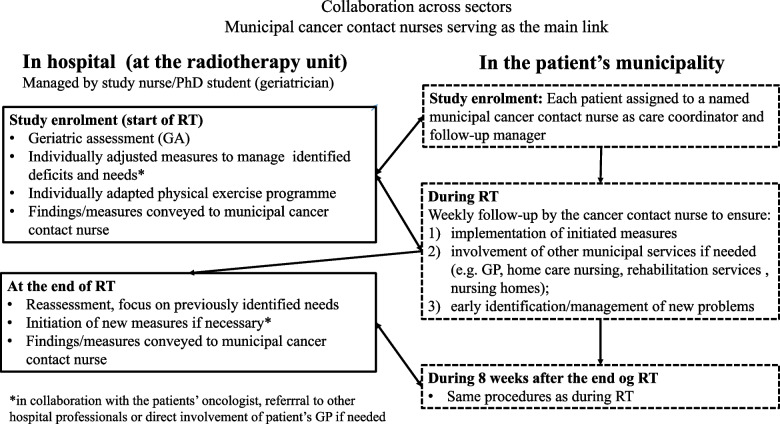
Collaboration across sectors. Municipal cancer contact nurses serving as the main link. *In collaboration with the patients’ oncologist, referrral to other hospital professionals or direct involvement of the patient’s GP if needed

First, the study nurse (Centre 1) and the PhD student (Centre 2) initiated each patient`s intervention at the hospital outpatient clinic. They performed a GA at the start of RT with a limited re-assessment at the end of RT, and in collaboration with the patients’ oncologist, they made a management plan targeting identified impairments (Fig. 2, Table 1). As part of this plan, the patients received an individually adapted physical exercise program.

**Table 1:** An overview over all pre-scheduled assessments in the geriatric assessment with management (GAM) intervention

In-hospital — at the study centres, managed by the study nurse/PhD student (geriatrician)				In the municipality by the cancer contact nurse	
**Geriatric assessment — at the start of RT and intervention**		**Prescheduled repeat at the end of RT**	**In-hospital assessor, at the start and repeat**	**Pre-scheduled minimum follow-up during RT and 8 weeks after RT**	
Domain and method				Every week (telephone)	Week 4 after RT (house call)
**Somatic health**					
Extra lab-test (HbA1c: glycated haemoglobin, FT4: free thyroxine 4, THS: thyroid stimulating hormone, vitamin B12)^a^		-	Hospital laboratory	**-**	**-**
Blood pressure		-	Study nurse/PhD student in patient consultation	**-**	**-**
Overall frailty	*Edmonton Frail Scale (EFS), scored 0 (fit) to 17 (severe frailty)*[26]^b^	EFS		-	EFS
Symptoms	Edmonton Symptom Assessment System (ESAS) [27]^c^	ESAS	Patient reported	ESAS — patient reported	ESAS — patient reported
Comorbidity	*Charlson comorbidity index (scored 0–26)* [28]^b,d^	_	Study nurse/PhD student, based on medical journal, information from oncologist and patient	-	-
	Old Americans’ Resource Survey (OARS) (scored 0–15) [29]^d^	_		-	-
Medications	Number, dose, and type of drug according to the Anatomical Therapeutic Chemical (ATC) System	All medications		-	All medications
Nutritional status	Mini Nutritional Assessment Short form (MNA-SF), scored 0 (worse) to 14 (better) [30]	MNA	Study nurse/PhD student in patient consultation	-	MNA-SF
	*Weight, height, weight loss*^b^	Weight		Weight — patient reported	Weight
**Mental health**		-			
Cognition	*Mini-Cog, scored 0 (worse) to 5 (better)* [31]^b^		Study nurse/PhD student	-	**-**
Depression	Geriatric Depression Scale (GDS)-15, scored 0–15 (higher scores, more symptoms) [32]	-	Patient reported in interview with study nurse/PhD student	-	**-**
**Function**					
Daily life activities	Lawton index, instrumental activities of daily living (IADL), scored 0 (fully dependent) to 8 (fully independent) (scores 0–8) [33] Barthel index, basic activities of daily living (ADL), scored 0 (fully dependent) to 20 (fully independent) [34]	-	Study nurse/PhD student in patient consultation	-	**-**
Mobility	*Short Physical Performance Battery (SPPB), scored 0 (worse) to 12 (better)*[35]^b^	-	Tested by study nurse/PhD student		**-**
	*Timed Up and Go (TUG), measured in seconds* [36]^b^	-			**-**
	*Grip strength *[37]^b^	-			**-**
	*Number of falls last 6 months, 0–1 vs* ≥ *2*^b^	-			**-**
**Social situation**					
*Civil status, living conditions, available help, home care, Oslo Social Support Scale* [38]		-	Study nurse/PhD student in patient consultation		

^a^Lab tests added to those routinely taken which normally include blood counts, serum-creatinine, serum-electrolytes, and serum liver enzymes

^b^Assessment marked in italics are assessments that were also performed at baseline in the control group to enable an appropriate comparison on central age geriatric domains

^c^Assesses 10 common cancer symptoms on scales ranging from 0 to 10, higher scores indicate more symptoms

^d^Higher scores indicate more comorbidity

Second, the management plan was the basis for a subsequent follow-up by a cancer contact nurse in the patient`s municipality. To ensure implementation of planned measures and adoptions to changing needs, the cancer contact nurse involved other primary care professionals when necessary, and followed the patient systematically during the overall intervention period. The follow-up included at least one weekly phone call with systematic symptom assessments, and a house call the fourth week after RT (Table 1).

Third, collaboration across sectors and coordination of services was a defined part of the intervention as smooth transitions and seamless trajectories of care remain a challenge [32, 33] (Fig. 2). For each patient, a named municipal cancer contact nurse was assigned the role as care-coordinator and a link between sectors. The study nurse and PhD student responsible for the initial GA conveyed the GA results and the management plan to the cancer contact nurse. Moreover, they were available during all working hours to facilitate contact between the municipal nurses and other hospital professionals if needed.

Pre-defined guidelines with detailed indications and suggestions for supportive measures in each GA domain were outlined as part of the intervention programme [25]. Further details on the task flow and systematic assessments included in the intervention are displayed in Fig. 2 and Table 1, respectively.

### Procedures

Baseline data were retrieved from the treating oncologists and electronic medical records (EMR) (including information on treatment intent), and through patient consultation/interview, testing, and self-report questionnaires. All assessments in the control and intervention group were performed by the study nurse and PhD student who managed the in-hospital part of GAM. In addition to ensure baseline status of pre-defined outcomes (assessed by QoL questionnaires and performance tests, see the “Outcomes and outcome assessment” section), the baseline assessments in both groups comprised number of falls in the last 6 months, comorbidities (Charlson Comorbidity Index [CCI]) [34], cognitive function (Mini-COG) [35], Timed Up and Go [36], and the Edmonton Frail Scale (EFS) scored 0–17 (fit 0–3, vulnerable 4–5, mild frailty 6–7, moderate frailty 8–9, severe frailty ≥ 10) [37] (Table 1). For the control group, the treating oncologist was blinded for the results unless severe, unrecognised health problems were revealed. For the intervention group, all assessments except answers to the QoL questionnaires were considered a part of the GA (Table 1).

Completion of QoL questionnaires was repeated at the end of RT and 4, 8, 16, 32, and 52 weeks later. Except at baseline and the end of RT, where the questionnaires were distributed by the study nurse/PhD student, the questionnaires were sent by post together with a pre-paid return envelope. If not returned within 2 weeks, the patients received one reminder. Physical performance was re-assessed 8 and 16 weeks after RT. This assessment was performed by trained physiotherapists in the patients’ municipalities who were otherwise not involved in the study, and thus blinded for the patient allocation.

### Outcomes and outcome assessment

The primary outcome was physical function (PF) reported by the patients on the European Organisation for Research and Treatment of Cancer Quality of life Questionnaire-C30 (QLQ-C30) [38], 8 weeks after RT completion. Secondary outcomes were overall QoL assessed by the EORTC QLQ-C30 global QoL scale and the EQ-5D-5L index [39], physical performance assessed by the Short Physical Performance Battery [SPPB)] [40], hand grip strength, and use of health care services and their costs. Symptom scores (fatigue-, pain-, dyspnoea-, sleeping disturbances-, loss of appetite) and emotional function from the EORTC QLQ-C30 were additional pre-defined outcomes.

The EORTC QLQ-C30 is a 30-item questionnaire comprising five functioning scales, a global QoL scale, and nine symptom scales/items [38]. All items are scored from 1 (not at all) to 4 (very much), except for the two items of the global QoL scale, which are scored from 1 (very poor) to 7 (excellent). Before analyses, raw scores are converted to scales ranging 0–100. Higher scores indicate better function on the global QoL- and functioning scales, and more symptoms on the symptom scales/items [41]. A difference of ≥ 10 points on any scale is considered clinically significant [42]. The EQ-5D-5L is a generic questionnaire measuring five dimensions of QoL/health (mobility, self-care, usual activities, pain/discomfort, and anxiety/depression) on five levels. The EQ-5D-5L-index was calculated by assigning values from the UK time-trade-off tariff to the different health states as generated [43, 44]. Values range from 1 = full health to 0 = dead, but values below 0 are possible, indicating states deemed worse than dead [43, 44]. The minimally clinically important difference (MCID) for this index is reportedly 0.08–0.1 [45, 46]. The SPPB assesses standing balance, walking speed, and ability to rise from a chair. The total score ranges from 0 to 12; high scores suggest better mobility [40]. Hand grip strength was measured in kilogrammes using a dynamometer [47], with an MCID reportedly being 5 to 6.5 kg [48].

Use of health care services from inclusion to 52 weeks after RT was retrieved for each patient from official Norwegian registries (the Norwegian Control and Payment of Health Reimbursements Database [KUHR] [49], the Norwegian Patient Registry [50] and the participating municipalities. Costs were calculated by multiplying service volume by a unit cost and summarising over service categories. GAM costs related to the work of the study nurse/PhD student and the municipal cancer contact nurse were stipulated by multiplying time spent by wage cost per hour. Further details on cost assessments and estimations are provided in Additional file 2 [49–52]. The date of death was extracted from the patient’s EMR.

To evaluate the GAM process, the study nurse and PhD student kept log notes of the GA results and the implemented measures. The municipal cancer contact nurses registered their involvement through weekly log-notes and questionnaires addressing tasks performed and time used (Table 1). Adherence to the in-hospital programme was retrospectively evaluated by exploring whether supportive measures were registered in these logs and implemented in accordance with GA findings and pre-defined guidelines. The municipal part was evaluated by briefly exploring the cancer contact nurses’ compliance to the weekly symptom registrations that were scheduled during the last part of the intervention period, i.e. from the end of RT to 8 weeks later.

### Sample size

The sample size calculation was tailored to a cluster-randomised design with 32 clusters and longitudinal analysis of covariance (ANCOVA) as the approach [25]. Assuming an intra-cluster correlation coefficient of 10%, standard deviation (SD) of 24 in each group, and correlation between baseline and follow-up measurement of 0.5, a total of 53 patients distributed in 16 clusters (proportionally to cluster size) in each group was needed to detect a difference of 12 points in the physical function EORTC QLQ-C30 scale at week 8 at the significance level of 5% with a power of 80%. Assuming an attrition of about 15–20% at 16-week post RT, we aimed at including 162 patients, 81 in each group. By 10 months of recruitment, one municipal cluster had withdrawn, no patients were included from the additional four, and only one patient was included from four clusters expected to include at least two each. The sample size was thus re-calculated. Accounting for the reduced number of clusters and keeping other assumptions unchanged, 69 patients were required in each group, i.e. 93 in each group for a sample size of 186 when including estimated attrition.

### Statistical analysis

Due to the open cluster-randomised design with an inherent risk of selection bias [53, 54], we compared characteristics between the control and intervention group by Student’s t- and χ^2^-test, as appropriate [55]. The cluster effect on study cluster level in outcome variables was assessed by intra-class correlation coefficient (ICC). To assess the difference in PF between the groups 8 weeks after RT (primary outcome), and the difference in trend in PF up to 52 weeks after RT, we performed longitudinal ANCOVA by estimating a linear mixed model (LMM) with random effects for patients nested within study cluster and fixed effects for baseline values, time and interaction between time and group variable. A significant interaction would imply a difference between the groups in trend in outcome variables. Post hoc analyses were then performed to assess between-group differences at each time point. Similar models were estimated to analyse patient-centred secondary- and selected additional outcomes (symptoms and emotional function). Analyses were performed on the intention-to-treat principle. The analyses assessing outcomes 8 weeks after RT were first performed for patients responding both at this point as well as at baseline. To avoid possible bias due to missing values in outcomes, and thus patients excluded from the analyses, sensitivity analyses with missing values imputed by LMM were conducted. Finally, for entirely explorative purposes, all analyses were repeated adjusting for co-variates likely to influence patient-centred outcomes, i.e., treatment intent, frailty status in terms of EFS scores in addition to age and gender.

To investigate the impact of the intervention on health care costs, LMMs with random effects for the municipality and fixed effects for group and treatment intent were estimated using log-transformed dependent cost variables due to a skewed distribution. Both unadjusted models and models adjusting for other potential cost drivers, i.e., age, gender, EFS score, and treatment intent, were estimated. For details, please see Additional file 2: Methods for the evaluation of the use and costs of health care services.

All tests were two-sided and results with *p*-values < 0.05 were considered statistically significant. The analyses were performed in STATA v16.

### Ethics and approval

The study was approved by the Regional Committee for Medical Research Ethics, South East Norway (ref. number 2018/2515), and was registered at ClinicalTrials.gov (NCT03881137).

## Results

### Patients

We enrolled a total of 178 patients, 89 in each group, representing 28 out of 32 randomised clusters and 34% of all patients registered as eligible (see Fig. 1 for details). Recruitment was stopped when preliminary estimates indicated that the primary outcome was completed for a required number of patients in both groups (more than 69 patients). Six control and five intervention clusters were represented by only one or two patients.

The mean age in the overall cohort was 74.1 (SD 5.4) years, and 38.2% were female. The most frequent cancer type was prostate cancer (42.7%), 91.5% had Eastern Cooperative Oncology Group Performance stats (ECOG PS) 0–1, the mean EFS score was 3.4, and 43.9% were vulnerable or frail (Table 2). The control and intervention groups were comparable with a few important exceptions. More patients in the intervention group received RT with curative intent, 76.4% versus 61.8% in the control group (*p* = 0.035), and consequently had a RT regimen with a longer duration and larger total irradiation dose (Table 2). Additionally, patients in the intervention group reported significantly better baseline scores on physical function (PF) (*p* = 0.022), dyspnoea (DY) (*p* = 0.033) and the EQ-5D-5L index (*p* = 0.029) (see Additional file 3: Table S3).

**Table 2:** Patient characteristics

		All patients(*n* = 178)	Control group(*n* = 89)	Intervention group(*n* = 89)	*p*-value
**Sociodemographic characteristics**					
**Age**					
Mean (SD)		74.1 (5.4)	74.0 (5.8)	74.2 (5.1)	0.848
**Sex**, *n* (%)					0.758
Female		68 (38.2)	35 (39.3)	33 (37.1)	
**Marital status**, *n* (%)					0.197
Married/cohabiting		122 (68.5)	65 (73.0)	57 (64.0)	
Single/divorced/widow(er)		56 (31.5)	24 (27.0)	32 (36.0)	
**Home care nursing**, *n* (%)		20 (11.2)	11 (12.4)	9 (10.1)	0.635
**Information on cancer disease and performance status**					
**Cancer type**, *n* (%)					0.665
Prostate		76 (42.7)	34 (38.2)	42 (47.2)	
Breast		40 (22.5)	21 (23.6)	19 (21.3)	
Lung		19 (10.7)	10 (11.2)	9 (10.1)	
Other		43 (24.2)	24 (27.0)	19 (21.3)	
**Stage**^a^, *n* (%)					0.374
0^b^		3 (1.7)	1 (1.1)	2 (2.2)	
I		27 (15.2)	15 (16.9)	12 (13.5)	
II		40 (22.5)	16 (18.0)	24 (27.0)	
III		56 (31.5)	27 (30.3)	29 (32.6)	
IV		46 (25.8)	25 (28.1)	21 (23.6)	
Not applicable^c^		6 (3.4)	5 (5.6)	1(1.1)	
**ECOG PS**, *n* (%)^d^					0.062
0–1		162 (91.5)	78 (87.6)	84 (95.5)	
2–4		15 (8.4)	11 (12.4)	4 (4.5)	
**Information on radiotherapy regimen**					
**RT intent**, *n* (%)					**0.035**
Curative		123 (69.1)	55 (61.8)	68 (76.4)	
Palliative		55 (30.9)	34 (38.2)	21 (23.6)	
**RT duration, weeks**					**0.009**
Mean (SD)		4.0 (2.0)	3.6 (2.0)	4.4 (2.0)	
**Total radiation dose (gray)**					** < 0.001**
Mean (SD)		49.8 (17.1)	45.5 (17.4)	54.1 (15.7)	
**Dose per fraction (gray)**					0.748
Mean (SD)		2.9 (1.6)	2.9 (1.0)	2.9 (2.1)	
Patients completing treatment as planned, *N* (%)		172 (96.6)	86 (96.6)	86 (96.6)	1.000
**Geriatric measures applied in both groups for comparison**					
EFS score (missing 1), ^e^ mean (SD)		3.4 (2.5)	3.6 (2.7)	3.3 (2.3)	0.582
EFS ≤ 3 (fit), *n* (%)		101 (57.1)	48 (54.5)	53(59.6)	
EFS 4–5 (vulnerable), *n* (%)		37 (20.9)	20 (22.7)	17 (19.1)	
EFS 6–7 (mild frailty), *n* (%)		28 (15.8)	14 (15.9)	14 (15.7)	
EFS 8–9 (moderate frailty), *n* (%)		7 (4.0)	2 (2.3)	5 (5.6)	
EFS ≥ 10 (severe frailty), *n* (%)		4 (2.3)	4 (4.5)	0 (0)	
Fall the last 6 months					0.515
0–1, *n* (%)			83 (93)	85 (96)	
≥ 2, *n* (%)			6 (7)	4 (4)	
Body mass index, mean (SD)		27.4 (4.7)	26.8 (4.6)	27.9 (4.6)	0.119
CCI,^f^ mean (SD)		1.2 (1.5)	1.2 (1.6)	1.3 (1.4)	0.611
Mini-Cog (missing 1),^g^ mean (SD)		4.1 (1.1)	4.3 (1.0)	3.9 (1.3)	0.071
TUG (missing 6),^h^ mean (SD)		10.2 (3.6)	10.2 (3.5)	10.2 (3.7)	0.927
Grip strength (missing 1),^h^ mean (SD)		32.0 (10.4)	31.5 (10.8)	32.6 (10.1)	0.488
SPPB (missing 1), ^i^ mean (SD)		9.9 (2.5)	9.7 (2.8)	10.1 (2.1)	0.234
**Survival**					
Deceased during study period (%)		25 (14.4)	11 (12.4)	14 (15.7)	0.454

^a^Stage according to the TNM classification

^b^Ductal carcinoma in situ receiving RT regimen similar to patients’ with invasive breast cancer stage I

^c^Patient with myelomatosis where the TNM classification does not apply

^d^ECOG PS = Eastern Cooperative Oncology Group Performance Status

^e^EFS = Edmonton Frail Scale, scores 0–17, scores 0–3 considered as fit, higher scores indicate increasing levels of frailty

^f^CCI = Comorbidity Index, scored 0–26, higher scores indicating more comorbidities

^g^Mini-Cog, screening of cognitive function, scored 0 (worse) to 5 (better)

^h^TUG = Timed Up and Go measured in seconds, higher numbers indicate poorer mobility; ^h^ Hand grip strength in kilogrammes, measured by a dynamometer

^i^SPPB = Short physical performance battery, scored 0–12, higher scores indicate better physical performance

### Survival and compliance

A total of 25 (14.4%) patients, 11 (12.4%) in the intervention group and 16 (18.0%) in the control group died within 52 weeks after RT (Fig. 1). Among patients alive at each assessment, compliance in completing the EORTC QLQ-C30 was 90% or more and largely similar in the two groups throughout follow-up. For the performance tests, only about $\frac34$ and $\frac23$ of the patients still alive in both groups were tested 8 and 16 weeks after RT, respectively (for details, see Additional file 4: Table S4).

### Adherence to the intervention programme and use of health services

Applying the pre-defined guidelines for when to implement supportive measures, the baseline prevalence of needs/problems within each GA domain ranged from 7% (depressive symptoms) to 33% of the patients (any ESAS score > 4) (See Additional file 5: Table S5). According to log-notes, any measure was implemented for 31% to 100% of individual needs/problems. The lowest proportions concerned mild/moderate hypertension and problems related to other comorbidities, which were rarely noted. The highest proportions (81% to 100%) were registered for nutritional problems, depression, and cognitive or functional impairments. Most measures were undertaken by the project nurse/PhD student themselves and/or implied notification of the patients’ oncologists or GPs (see Additional file 5: Table S5). There were few referrals to other professionals. For patients surviving 8 weeks or more after RT (*n* = 86), the scheduled ESAS assessment was performed for 73% to 90% of patients each week (at mean 80%).

### Main outcomes, patient-centred

We found no significant differences between the intervention and control groups on any of the pre-defined primary (PF) and secondary patient-centred outcomes 8 weeks after RT, except for the EQ-5D-5L index (Table 3). This index declined from 0.83 to 0.80 in the intervention group in contrast to an observed improvement in the control group from 0.78 to 0.81. Both differences were below the reported MCID. Sensitivity analyses with imputation for missing values showed similar results for all outcomes, except for grip strength demonstrating a significantly larger increase in the intervention group as compared to the control groups (mean difference in change − 2.08 [95% − 2.71; − 1.45], *p* < 0.001), which was though below clinical significance [48]. When adjusting for treatment intent, EFS score, age and gender, the results of all analyses including the sensitivity analyses remained the same as in the unadjusted ones (Table 3). In all outcomes, cluster effect on study cluster level was present according to ICC but did not affect the results.

**Table 3:** Comparison of patient-centred outcomes between study groups, 8 weeks after termination of radiotherapy^a^

**Outcome**		**Group**	**Baseline**	**8 weeks**
			**Mean (SD)**	**Mean (SD)**
**Physical function**^**b**^				
ICC = 0.33		Control, *N* = 81	77.2 (23.2)	73.9 (25.7)
		Intervention, *N* = 76	84.7 (17.4)	81.3 (20.4)
Mean difference^**c**^				0.60 (− 3.02; 4.21)
* p*-value				0.747
Mean difference^**f**^				0.57 (− 3.00; 4.15)
* p*-value				0.753
**Global Quality of Life**^**b**^				
ICC = 0.23		Control, *N* = 81	71.0 (19.0)	68.0 (21.7)
		Intervention, *N* = 76	77.3 (20.3)	74.1 (21.0)
Mean difference^**c**^				1.08 (− 3.09; 5.25)
* p*-value				0.611
Mean difference^**f**^				1.00 (− 3.18; 5.19)
* p*-value				0.639
**EQ-5D-5L index**^**d**^				
ICC = 0.15		Control, *N* = 87	0.78 (0.20)	0.81 (0.18)
		Intervention, *N* = 87	0.83 (0.17)	0.80 (0.21)
Mean difference^**c**^				− 0.06 (− 0.09; − 0.02)
* p*-value				0.002
Mean difference^**f**^				− 0.06 (− 0.10; − 0.03)
* p*-value				0.001
**SPPB**^**e**^				
ICC = 0.06		Control, *N* = 64	10.1 (2.3)	10.5 (2.3)
		Intervention, *N* = 62	10.4 (2.0)	10.8 (1.9)
Mean difference^**c**^				0.09 (− 0.33; 0.51)
* p*-value				0.672
Mean difference^**f**^				0.12 (− 0.30; 0.53)
* p*-value				0.581
**Grip strength**				
ICC = 0.26		Control, *N* = 64	32.8 (10.8)	33.7 (13.8)
		Intervention, *N* = 62	33.0 (10.2)	34.8 (9.2)
Mean difference^**c**^				0.93 (− 0.49; 2.35)
* p*-value				0.198
Mean difference^**f**^				0.95 (− 0.47; 2.37)
* p*-value				0.189

^a^Only patients with scores at both baseline and 8 weeks included

^b^Physical function and global quality of life, respectively, measured by the European Organisation for Research and Treatment of Cancer Quality of life Questionnaire-C30 (QLQ-C30)

^c^Estimated by longitudinal ANCOVA analysis, adjusted for baseline values, mean difference with 95% confidence intervals

^d^EQ-5D-5L index = EuroQoL-5 dimension-5 level index

^e^*SPPB* Short Physical Performance Battery

^f^Estimated by longitudinal ANCOVA analysis, adjusted for baseline values, intention, Edmonton frail scale, gender, and age, mean difference with 95% confidence intervals; ICC stands for intra-class correlation coefficient

**Fig. 3:**
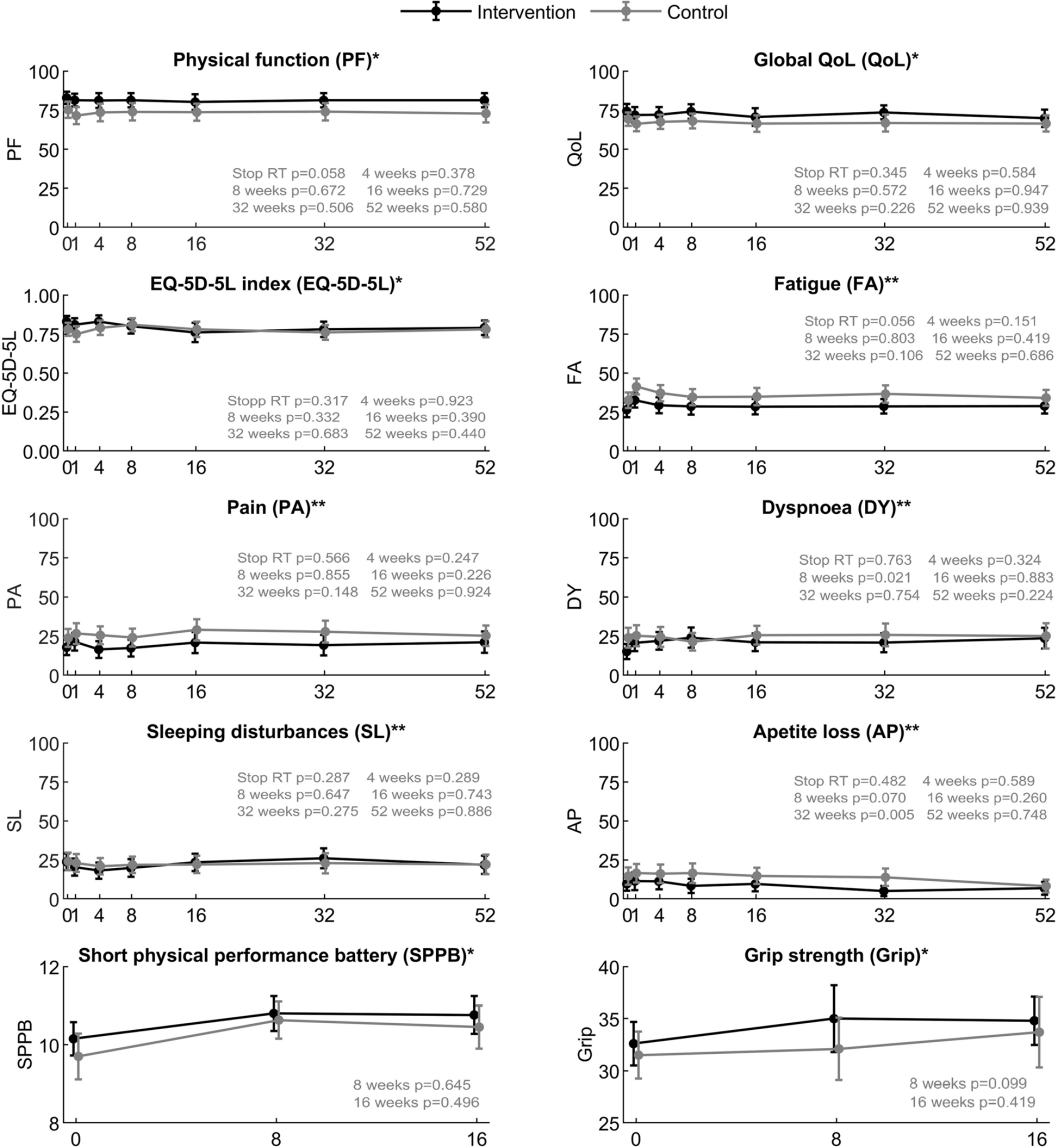
Results of longitudinal ANCOVA for between-group differences in trend in outcomes (unadjusted) and post hoc analyses assessing between-group differences at each time point presented as *p*-values. Curves showing mean scores with 95% confidence intervals at each assessment point for the two study groups, ^*^higher scores indicate better physical function, global QoL, health index, physical performance, or grip strength, respectively, ^**^higher scores indicate more symptoms

We found no difference in trend between groups (non-significant interaction terms) for PF, global QoL, SPPB scores, grip strength, fatigue (FA), pain (PA), sleeping disturbances (SL), dyspnoea (DY) (Fig. 3), and emotional function (data not shown). For the appetite loss (AP) and EQ-5D-5L, there was a significant difference between the groups in trend from RT stop (reference) to week 32 (*p* = 0.040) and from RT stop to week 8 (*p* = 0.039), respectively (Fig. 3). For AP the difference was in favour of the intervention group, whereas the difference for EQ-5D-5L favoured the control group. None was clinically significant. In the exploratory analysis adjusting for the pre-chosen confounders, a difference in trend in DY from RT stop to week 8, sleeping disturbances (SL) from RT stop to week 32, and EQ-5D-5L from stop RT to week 16 became significant (*p* = 0.035, *p* = 0.033, and *p* = 0.041, respectively). They were all in favour of the control group, and none reached clinical significance. Furthermore, the difference in trend in AP from RT to week 32 became non-significant after adjustment (Fig. 3). Post hoc analyses for both the adjusted and unadjusted analyses revealed a few between-group differences at specific time points, none of which reached clinical significance (Fig. 3).

### Use and costs of health services

There were no significant differences in the use of hospital and primary care services between the groups, except for fewer in-hospital days and more outpatient visits in the intervention group during the RT period (Table 4). The scheduled intervention (the in-hospital GA, implementation of targeted measures, and the municipal follow-up) resulted in a mean of 7.3 (SD 4.8) nursing hours per patient and a mean cost of 350€ (SD 222 €). This represented 5% of the total costs for health care services during the intervention period and 3% during the whole study period. Including this and all other costs, no statistically significant difference in cost between the intervention and the control group was found, neither by unadjusted analyses nor when adjusting for other potential cost drivers (Table 5). Factors associated with higher costs were palliative treatment intention, being male, and having higher frailty scores, where one point increase in the Edmonton Frail Scale resulted in 9.4% higher costs (Table 5). Crude cost data are displayed in Table 4.

**Table 4:** Volume and costs of health care services used by each study group during study participation

	All participants *N* = 178	Intervention cohort *N* = 89	Control cohort *N* = 89	*p*-value
	Mean (SD)	Mean (SD)	Mean (SD)	
**Volume of services**				
During the intervention period^a^				
Specialist services				
• In-hospital days	1.1 (3.7)	0.4 (1.9)	1.9 (4.8)	**0.011**
• Visits to outpatient clinics	23.3 (12.1)	25.8 (11.8)	20.7 (12.0)	**0.004**
Primary health care services				
• GP, visits	1.33 (1.6)	1.5 (1.6)	1.2 (1.7)	0.238
• Physiotherapy, visits	0.2 (1.0)	0.2 (1.0)	0.2 (1.0)	0.883
• Home nursing, hours	0.5 (2.0)	0.4 (1.6)	0.6 (2.4)	0.381
• Rehabilitation, hours	0.0 (0.2)	0.0 (0.2)	0.0 (0.2)	0.857
• Nursing home stay, days	0.1 (1.3)	0.3 (1.9)	0	0.179
During the post-intervention period^b^				
Specialist services				
• In-hospital days	5.1 (9.7)	4.7 (10.3)	5.4 (9.1)	0.633
• Visits to outpatient clinics	10.3 (10.7)	10.6 (11.9)	9.9 (9.4)	0.671
Primary health services				
• GP, visits	16.0 (12.3)	17.6 (13.5)	14.5 (10.9)	0.089
• Physiotherapy, visits	4.1 (11.4)	5.3 (13.0)	3.0 (9.6)	0.177
• Home nursing, hours	12.3 (49.2)	9.1 (52.3)	15.6 (45.9)	0.376
• Rehabilitation, hours	1.5 (10.6)	2.2 (14.7)	0.9 (3.3)	0.402
• Nursing home stay, days	3.4 (12.9)	2.9 (12.3)	4.0 (11.8)	0.527
Hours of GAM^c^ intervention		7.3 (4.8)		
• In-hospital		2.0 (0)		
• Municipal cancer contact nurse		5.3 (4.7)		
**Cost of services**^d^				
Costs of GAM^c^ intervention		350 (222)		
Cost during intervention period	7362 (527)	7016 (4162)	7709 (5865)	0.382
• Specialist health care	5871 (3998)	5633 (3613)	6109 (4341)	0.428
• Primary health care	1316 (2268)	1033 (1704)	1601 (2854)	0.109
Cost during post-intervention period	5504 (8747)	4815 (8108)	6192 (9338)	0.295
• Specialist health care	2729 (6029)	2315 (4329)	3142 (7351)	0.362
• Primary health care	2775 (2904)	2500 (6426)	3050 (4888)	0.521
Costs during whole study period, mean (SD)^e^	12,866 (11,715)	11,831 (10,759)	13,901 (12,574)	0.240
• Specialist health care	8599 (8099)	7984 (6562)	9250 (9382)	0.285
• Primary health care	4092 (7284)	3533 (7621)	4651 (6929)	0.307

^a^Time from inclusion to 8 weeks after termination of radiotherapy

^b^time from 8 to 52 weeks after radiotherapy

^c^*GAM *geriatric assessment with management

^d^all cost in Euro and 2020 prices

^e^time from inclusion up to 52 weeks after termination of radiotherapy

**Table 5:** Comparison of costs of health care services between study groups^a^

Variable	Unadjusted models		Adjusted model	
	RC^b^ (95% CI^c^)	*p*-value	RC^b^ (95% CI^c^)	*p*-value
**Costs during the whole study period**^**d**^ (ICC^**g**^ = 0.05)				
Intercept			12.65 (11.45; 13.85)	**< 0.001**
Group, intervention	− 0.09 (− 0.29; 0.11)	0.365	− 0.02 (− 0.19; 0.15)	0.827
Intention, curative	− 0.59 (− 0.79; − 0.39)	**< 0.001**	− 0.39 (− 0.60; − 0.19)	**< 0.001**
Edmonton frail scale	0.11 (0.08; 0.15)	**< 0.001**	0.09 (0.05; 0.13)	**< 0.001**
Gender, female	− 0.20 (− 0.41; − 0.0002)	**0.050**	− 0.19 (− 0.36; − 0.007)	**0.041**
Age	− 0.005 (− 0.02; 0.01)	0.618	− 0.01 (− 0.03; 0.002)	0.087
**Costs during the intervention period**^e^ (ICC^**g**^ = 0)				
Intercept			11.99 (10.91; 13.06)	**< 0.001**
Group, intervention	− 0.03 (− 0.21; 0.14)	0.717	− 0.01 (− 0.17; 0.15)	0.897
Intention, curative	− 0.26 (− 0.45; − 0.07)	**0.007**	− 0.03 (− 0.21; − 0.16)	0.759
Edmonton frail scale	0.09 (0.06; 0.13)	**< 0.001**	0.10 (0.06; 0.13)	**< 0.001**
Gender, female	− 0.32 (− 0.49; − 0.14)	**< 0.001**	− 0.33 (− 0.49; − 0.17)	**< 0.001**
Age	− 0.005 (− 0.02; 0.01)	0.537	− 0.01 (− 0.03; − 0.0003)	**0.045**
**Costs during the post-intervention period**^f^ (ICC^**g**^ = 0)				
Intercept			15.61 (11.47; 19.75)	** < 0.001**
Group, intervention	− 0.28 (− 0.89; 0.33)	0.363	− 0.22 (− 0.82; 0.38)	0.474
Intention, curative	− 0.34 (− 1.00; 0.32)	0.308	− 0.24 (− 0.95; 0.47)	0.515
Edmonton frail scale	0.05 (− 0.07; 0.17)	0.423	0.06 (− 0.07; 0.19)	0.376
Gender, female	0.19 (− 0.43; 0.82)	0.545	0.15 (− 0.46; 0.77)	0.625
Age	− 0.07 (− 0.13; − 0.02)	**0.011**	− 0.08 (− 0.13; − 0.02)	**0.007**

^a^ Results of linear mixed models for LN (natural logarithm)-transformed costs, with adjustment for cluster effects on the municipal level, when necessary according to Bayes Information Criterion

^b^*RC* regression coefficient

^c^*CI* confidence interval

^d^time from inclusion up to 52 weeks after termination of radiotherapy

^e^time from inclusion to 8 weeks after termination of radiotherapy

^f^time from 8 to 52 weeks after radiotherapy

^g^*ICC* intra-class correlation

## Discussion

In this pilot cluster RCT targeting older patients receiving RT with palliative or curative intent, we found no significant impact of a GAM intervention on either patient-reported physical function, overall QoL, physical performance, symptoms, or use and costs of health services.

To the best of our knowledge, this is the first controlled study evaluating a GAM intervention solely in an RT setting, and the first to include collaboration between specialist and primary health care. Opposed to our findings, the majority of RCTs from surgical and medical settings have shown a positive impact of GAM on at least some treatment outcomes [10, 11]. The most consistent benefits seem to be on toxicity, complications, and treatment completion [10, 11, 13, 15, 56, 57, 58, 59]. QoL, function, and symptoms have been inconsistently included and assessed. In line with our results, three previous RCTs found either no effect on QoL and functional limitations [16] or no effect on daily life activities and physical performance [15, 60], except for fewer falls [15]. Contradictory, four studies reported either a positive impact on QoL (functional aspects) [12], less decrement in QoL and reduced severity of symptoms [14], or improvement in a few QoL aspects [13, 61]. Thus, further research is warranted to clarify what may be achieved on QoL and function by GAM in oncology settings.

In our cost analyses, we identified palliative treatment intent, poorer frailty scores, and gender as cost-driving factors in accordance with existing knowledge [62, 63, 64]. We found no impact of the GAM intervention. Our results coincide with one previous RCT reporting direct cost estimates of GAM [65], and partly contradict another more recent one, showing a cost-saving effect in a curative, but not in a palliative chemotherapy setting [19]. Thus, firm evidence of a cost-saving effect in older patients with cancer remains to be found. However, based on present results and a range of studies looking at the impact on length and number of hospital stays, there are no indications that the use of hospital services and subsequently costs may increase [10, 11].

### Methodological considerations

Drawing general conclusions regarding the outcomes of GAM, based on existing evidence, is seriously hampered by substantial differences in the choice of outcomes, assessment methods, study populations, and intervention designs [10, 11, 66]. These are all imperative factors for the interpretation of results, along with an understanding of the study context and the comparative conventional care, which is often poorly described [66]. The present study was designed as a pilot study to evaluate a complex intervention, including study design, patient selection, and feasibility, and to provide an estimate of a potential effect size. There are several study strengths that we find should be preserved in a future RCT. The initial GA was performed with established methods covering recommended domains [7] and with pre-planned guidelines for the management of identified problems/needs [10]. The intervention addressed major challenges in the care for older patients with cancer, i.e. care coordination and systematic monitoring [67]. We chose outcomes reflecting clinical benefits that are highly prioritised in older age [68, 69], and demonstrated excellent compliance using well-validated patient-reported outcomes measures (PROMs) [70]. Finally, both specialist and primary care registries were included in the health economic evaluation.

However, several study limitations may explain the lack of intervention effect. We included patients irrespective of frailty status, and opposed to most studies, set the lower age limit to 65 years in line with the ASCO Guideline [71]. A selection of the fitter patients may have occurred, particularly in the intervention group, as a majority of our study sample turned out to be fit. This may have diluted the intervention effect [16]. The heterogeneity of the study sample in terms of cancer type, stage, and treatment intent may also have contributed since treatment burden (length, intensity, side effects) varies widely with these factors. Further, in contrast to recent recommendations [10], the GA results were not accounted for in the oncological decision-making. The intervention was initiated by single professionals, largely dependent on the action of others (patients’ oncologist, GP, or cancer contact nurse) to effectuate most supportive measures. No difference between groups in the use of primary health care services may be related to fit patients (no need for services), but it can also indicate that identification and/or management of problems/needs might have failed, resulting in minor contrast to conventional care. The effectiveness of the municipal part of the intervention, including competence in identifying geriatric problems, may therefore be questioned. We expect that a pending, pre-planned evaluation of the intervention`s feasibility, including interviews with patients and nurses [25], will answer these questions.

Further study limitations include the coinciding COVID-19 pandemic, which seriously affected the study conduct. The recruitment had to be paused for several months, and a heavy, extra workload was experienced by health services including the cancer contact nurses. Non-intended interventions in the control group may also be considered since baseline assessments were performed by the same staff who initiated the intervention. Finally, it must be kept in mind that being a pilot study, it was not dimensioned for either sub-group analyses or detailed cost-assessments of service offers of low frequency.

## Conclusions

In this controlled pilot study on GAM applied in collaboration between hospital- and primary care services in an RT setting, no improvement of patients’ global QoL, function, or symptom burden could be revealed, nor did it affect health care utilisation and costs. Potential causes for this lack of effect represent important learning points for future studies that are needed to define the role and organisation of GAM in RT settings. Our main recommendations are:

- Target the patients likely to benefit from the intervention, i.e. those with pre-frailty and frailty.
- Keep the study sample homogeneous in terms of cancer type and treatment intent.

Based on recent developments in geriatric oncology, the intervention should be strengthened by taking the initial GA results into account in oncological decisions, and by integrating geriatric expertise into older patients’ treatment teams to efficiently manage any geriatric impairments. Finally, we advocate further use of precisely defined PROMs to capture true benefits for the patients.

### Supplementary Information

Additional file 1: Table S1. Conventional Care. Established routines and services available to all study participants.

Additional file 2. Methods for the evaluation of the use and costs of health care services: Comparison of the intervention and control group. Price list of Norwegian specialist and primary health care services included (Table S2).

Additional file 3: Table S3. Baseline quality of life scores for the overall cohort and according to treatment groups.

Additional file 4: Table S4. Patients alive and compliance in completing questionnaires and physical performance tests at each scheduled assessment point.

Additional file 5: Table S5. Initial GA results, prevalence of problems/needs in line with pre-defined guidelines and corresponding measures registered as implemented in log-notes.

## Data Availability

Due to a statement by the Data Protection Officer at Innlandet Hospital Trust, and in accordance with Norwegian privacy regulations, data cannot be shared publicly because they are confidential (due to the consent given by the participants when included in the study). It is possible to extract information, upon request. Proposals should be directed to the Research Department of Innlandet Hospital Trust; contact: SIHFDLforskning@sikt.sykehuspartner.no.

## Abbreviations

ADL: Activities of daily living
ANCOVA: Longitudinal analysis of covariance
AP: Appetite loss as measured by the European Organisation for Research and Treatment of Cancer Quality of life Questionnaire-C30
ATC System: Anatomical Therapeutic Chemical System
BMI: Body mass index
CCI: Charlson Comorbidity Index
CGA: Comprehensive geriatric assessment
DY: Dyspnoea as measured by the European Organisation for Research and Treatment of Cancer Quality of life Questionnaire-C30
ECOG PS: Eastern Cooperative Oncology Group Performance
EFS: Edmonton Frail Scale
EORTC QLQ-C30: The European Organisation for Research and Treatment of Cancer Quality of life Questionnaire-C30
EQ-5D-5L: The EuroQual-5Dimension-5Llevel questionnaire
ESAS: Edmonton Symptom Assessment System
FA: Fatigue as measured by the EORTC QLQ-C30
GA: Geriatric assessment
GAM: Geriatric assessment with management
GDS: Geriatric depression scale
GlobalQoL: Global quality of life as measured by the EORTC QLQ-C30
GP: General practitioner
IADL: Instrumental activities of daily living
ICC: Intra-cluster correlation
LMM: Linear mixed model
MCID: Minimal clinically important difference
MNA-SF: Mini nutritional assessment – short form
PA: Pain as measured by the EORTC QLQ-C30
PF: Physical function as measured by the EORTC QLQ-C30 questionnaire
QoL: Quality of life
RCT: Randomised controlled trial
RT: Radiotherapy
SD: Standard deviation
SL: Sleeping disturbances as measured by the EORTC QLQ-C30
SPPB: Short physical performance battery
TUG: Timed up and go

## Declarations

### Declarations

Ethics approval and consent to participate.

This study was approved by the Regional Committee for Medical Research Ethics, South East Norway (ref. number 2018/2515), and was registered at clinicaltrials.gov (ID NCT03881137). All participants provided written informed consent.
