# Supplementary material for: Geriatric assessment with management for older patients with cancer receiving radiotherapy: a cluster-randomised controlled pilot study
Source: BMC Med. 2024 Jun 10;22:232. doi: 10.1186/s12916-024-03446-4 (PMC11163782; doi:10.1186/s12916-024-03446-4)
Supplement: Supplementary file 1 — Additional file 1: Table S1. Conventional Care. Established routines and services available to all study participants. [file 12916_2024_3446_MOESM1_ESM.docx]

**Additional file 1**

**Additional file 1: Table S1.** Conventional Care. Established routines and services available to all study participants

| **General: specialist services** | |
| --- | --- |
| - Hospital inpatient services, free of charge | |
| - Hospital outpatient services with limited co-payment ^a^ | |
| - Consultations with specialists (public) outside hospital, limited co-payment ^a^ | |
| - Rehabilitation in- and outpatient services (outpatient services with co-payment) | |
| **General: primary care services** | |
| - Family GP available for the vast majority ^a^ | |
| - Home care nursing, free of charge | |
| - Practical help at home, limited co-payment relative to income | |
| - Physiotherapist and social workers generally available, limited co-payment ^a^ | |
| - Nutritionist, and occupational therapists not available in all municipalities | |
| - Nursing homes, co-payment relative to income | |
| **Specific: specialist services related to radiotherapy (RT) or the RT centres** | |
| **Centre 1, local hospital** | **Centre 2, university hospital** |
| RT provided as external beam irradiation, according to national guidelines, regimens given in daily doses (on weekdays) for a few days to several weeks targeting the primary tumour and/or metastases and/or surrounding tissue. Treatment with palliative intent conventionally has lower total doses compared to curative treatment | |
| RT routinely provided as outpatient treatment, hospital admission or admission to hospital hotel lodgings on demand | |
| Consultation with radiation oncologist at start and end of RT, no routines for geriatric screening or assessment | |
| Follow-up after treatment mainly by patients’ oncologist outside the RT centre or patients’ GP | |
| - Multiprofessional information assembly for patients receiving curative RT for prostate and breast cancer | ---------- |
| - Consultations with cancer nurse employed and located at the RT centre by start and end of RT | - No specific routines for consultation with cancer nurses and no cancer nurse specifically employed at the RT centre. Most patients having access to an assigned nurse at the hospital ordinary outpatient oncology clinic |
| - Fitness centre for patients with cancer ^b^ in the hospital building. Widely recommended by the cancer nurses at the RT centre. Physiotherapist available for personal supervision. No payment | - Fitness centre^b^ for patients with cancer in a neighbouring building. Physiotherapist available for personal supervision. No payment |
| Hospital based physiotherapists, occupational therapists, nutritionists, palliative care specialist and geriatrician/geriatric nurse available on demand/by referral from patients’ oncologist | |
| **Specific: primary health care – locally adapted services** | |
| - Cancer contact nurse available in all participating municipalities. Contact widely recommended by the cancer nurse at the RT centre, in particular for patients with advanced disease. | - No cancer contact nurse, but a central cancer co-ordinator not working with patients directly, but available for mediation of contact with relevant municipal health professionals |

^a^ Co-payment for prescribed medications, outpatient hospital treatment, GP consultations, and physiotherapy up to a yearly limit of about 230 Euros, ^b^ Fitness centre (“Breathing rooms”) organised by the non-profit organisation “Active against Cancer” (https://aktivmotkreft.no)
