# Supplementary material for: Geriatric assessment with management for older patients with cancer receiving radiotherapy: a cluster-randomised controlled pilot study
Source: BMC Med. 2024 Jun 10;22:232. doi: 10.1186/s12916-024-03446-4 (PMC11163782; doi:10.1186/s12916-024-03446-4)
Supplement: Supplementary file 2 — Additional file 2. Methods for the evaluation of the use and costs of health care services: Comparison of the intervention and control group. Price list of Norwegian specialist and primary health care services included (Table S2). [file 12916_2024_3446_MOESM2_ESM.docx]

**Additional file 2**

**Additional file 2. Methods for the evaluation of the use and costs of health care services: Comparison of the intervention and control group**

**Perspective and time horizon**

To examine the economic consequences of the GAM intervention, we have evaluated the costs of the intervention program itself and the costs of health care services received by the participants. We have applied a health service perspective and included costs for both primary and specialized health care services. In line with the evaluation of the patient centred outcomes, quality of life (QoL) and functioning, we have used a 52- week follow-up period.

**Data collection**

Primary health care: Services from GPs, municipal emergency centres, physiotherapy, occupational therapy, laboratory analyses initiated by the GP, patient transport, and visits to specialist not located in the hospitals’ outpatient clinics, were extracted from the Norwegian Control and Payment of Health Reimbursements Database (KUHR) (49). This register holds information on claims in addition to patient co-payments. In addition, data on home nursing care, home care services, institutional care, rehabilitation services, meals-on-wheels, and safety alarm, were collected from the service registries in the participating municipalities.

Specialist health care: In-hospital stays and visits to outpatient clinics were extracted from the Norwegian Patient Registry (50). This registry covers all publicly financed specialist health care services in Norway.

**Cost calculations**

The cost of the intervention programme included the in-hospital part of the GA performed by the project nurse and the PhD student, and municipal follow-up by the cancer contact nurse. For each patient, the in-hospital part of the GA was stipulated to take on average two hours. This included assessments at baseline and by the end of RT, and direct as well as indirect patient contact. Time spent by the municipal cancer contact nurse on activities during the follow up of each patient was logged by the nurse. Time spent was multiplied by wage cost per hour (see Table S2, below). In case of individually adapted physiotherapy, occupational therapy, etc., this was included in regular registries of primary care services (KUHR and NPR).

**Table S2:** Price list for intervention management, primary and specialist health care services. In Euros (2020).

| **Health care service** | **Unit** | **Unit cost in Euro** | **Source of information** | |
| --- | --- | --- | --- | --- |
| **Intervention management** |  |  | |  |
| Specialist nurse | Hour | 51 | | Average wage cost level for nurses at Study Centre 2 (the university hospital and in the surrounding city districts) |
| Municipal cancer contact nurse | Hour | 47 | |  |
| **Primary care services** |  |  | |  |
| Rehabilitation  (Physiotherapy, occupational therapy, rehabilitation for activities of daily living) | Hour | 85 | | Accounting centre, municipality surrounding Study Centre 2. |
| Home care nursing | Hour | 83 | |  |
| Practical help at home | Hour | 81 | |  |
| Nursing home, short-term stay | Night | 279 | |  |
| Nursing home, long-term stay | Night | 272 | |  |
| Day care centre | Day | 72 | |  |
| Meals on wheels | Month | 158 | |  |
| Safety alarm | Month | 4 | |  |
| **Specialist services** |  |  | |  |
| Hospital in-patient stays | Stay | DRG-weights and national unit cost 2020 | | Norwegian Directory of Health. DRG system (51) |
| Hospital out-patient visits | Visits | DRG-weights and national unit cost 2020 | |  |

Costs of primary care and specialist services were calculated by multiplying service volumes by a unit cost and summarizing over service categories. The included service categories, the accompanying unit costs and source of information are listed in Table S2, above.

Specialist health care costs were calculated from the national activity based financing system, where each stay and visit is assigned a Diagnosis-Related Group (DRG) and the accompanying cost-weight (51). The DRG-based costs are expected to cover the average costs of a treatment in Norway, but for outpatient visits the patients’ co-payments must be added. For primary health care services covered by KUHR costs were based on claims and out-of-pocket payments. Costs were calculated in 2020 Euros (1 Euro=10.73 NOK; mean value for 2020) (52).

**Statistics**

By estimating three linear mixed models with fixed effects for group and treatment intent, we investigated the impact of the intervention on health care costs for three periods of time: 1) the overall study period, 2) the intervention period from inclusion to eight weeks after the end of RT, and 3) the post-intervention period from eight to 52 weeks after the end of RT. An interaction between the group (control versus intervention) and treatment intent (palliative versus curative) was assessed but not present. Random effects for the municipality were included in the models when relevant according to the Bayes Information Criterion. Due to a skewed distribution of the dependent variables these were log-transformed for the regression analyses. Next, to explore the impact of other potential cost drivers, the three models were adjusted for age, gender, frailty score, and treatment intention.
