# Supplementary material for: Geriatric assessment with management for older patients with cancer receiving radiotherapy: a cluster-randomised controlled pilot study
Source: BMC Med. 2024 Jun 10;22:232. doi: 10.1186/s12916-024-03446-4 (PMC11163782; doi:10.1186/s12916-024-03446-4)
Supplement: Supplementary file 3 — Additional file 3: Table S3. Baseline quality of life scores for the overall cohort and according to treatment groups. [file 12916_2024_3446_MOESM3_ESM.docx]

Additional file 3

**Additional file 3: Table S3.** Baseline quality of life scores for the overall cohort and according to treatment groups.

| **Scales/scores** | **All patients** | **Control group** | **Intervention group** | **p-value** |
| --- | --- | --- | --- | --- |
|  | N (%) | N (%) | N (%) |  |
| **QLQ-C30 functioning scales^a^** | |  |  |  |
| Physical function (PF) | 79.0 (22.3) | 75.2 (24.7) | 82.9 (18.8) | **0.022** |
| Global QoL (QL) | 71.7 (21.7) | 69.3 (20.7) | 74.2 (22.6) | 0.132 |
| Role functioning (RF) | 77.9 (28.6) | 74.0 (31.6) | 82.0 (24.7) | 0.062 |
| Emotional functioning (EF) | 86.9 (16.3) | 86.2 (15.7) | 87.6 (17.0) | 0.569 |
| Cognitive functioning (CF) | 86.0 (15.5) | 86.3 (15.4) | 85.6 (15.7) | 0.766 |
| Social functioning (SF) | 78.4 (26.1) | 77.0 (26.1) | 79.9 (26.2 | 0.460 |
| **QLQ-C30 symptom scales/scores^b^** | |  |  |  |
| Fatigue (FA) | 29.5 (23.5) | 32.5 (24.2) | 26.4 (22.6) | 0.090 |
| Nausea and vomiting (NV) | 5.3 (13.7) | 5.1 (11.3) | 5.6 (15.8) | 0.810 |
| Pain (PA) | 20.9 (25.9) | 23.8 (27.6) | 17.8 (23.7) | 0.128 |
| Dyspnea (DY) | 19.6 (27.5) | 24.0 (30.6) | 15.1 (23.2) | **0.033** |
| Insomnia (SL) | 23.9 (26.4) | 24.0 (27.1) | 23.8 (25.9) | 0.957 |
| Appetite loss (AP) | 12.1 (24.0) | 14.6 (26.6) | 9.6 (20.9) | 0.165 |
| Constipation (CO) | 14.9 (25.2) | 17.8 (28.6) | 11.9 (20.9) | 0.120 |
| Diarrhea (DI) | 18.1 (25.4) | 20.5 (26.0) | 15.7 (24.8) | 0.218 |
| Financial difficulties (FI) | 5.3 (16.6) | 3.7 (13.7) | 6.9 (19.1) | 0.210 |
| **EQ-5D-5L** |  |  |  |  |
| EQ-5D-5L index**^c^** | 0.806 (0.188) | 0.775 (0.210) | 0.837 (0.157) | **0.029** |

^a^ higher scores indicate better functioning; ^b^ higher scores indicate more symptoms; ^c^ higher scores indicate better health/quality of life
