# Supplementary material for: Geriatric assessment with management for older patients with cancer receiving radiotherapy: a cluster-randomised controlled pilot study
Source: BMC Med. 2024 Jun 10;22:232. doi: 10.1186/s12916-024-03446-4 (PMC11163782; doi:10.1186/s12916-024-03446-4)
Supplement: Supplementary file 4 — Additional file 4: Table S4. Patients alive and compliance in completing questionnaires and physical performance tests at each scheduled assessment point. [file 12916_2024_3446_MOESM4_ESM.docx]

Additional file 4

**Additional file 4: Table S4.** Compliance in completing questionnaires and physical performance tests at each scheduled assessment point

|  | | **Baseline** | | **Stop RT** | | **4 weeks** | | **8weeks** | | **16weeks** | | **32weeks** | | **52weeks** | |
| --- | --- | --- | --- | --- | --- | --- | --- | --- | --- | --- | --- | --- | --- | --- | --- |
|  | | I | C | I | C | I | C | I | C | I | C | I | C | I | C |
| Patients alive (n) | | 89 | 89 | 89 | 89 | 89 | 89 | 86 | 88 | 85 | 82 | 81 | 77 | 79 | 73 |
| Patients diseased (n) | | 0 | 0 | 0 | 0 | 0 | 0 | 3 | 1 | 4 | 7 | 8 | 12 | 10 | 16 |
| QLQ-C30 compliance | |  |  |  |  |  |  |  |  |  |  |  |  |  |  |
|  | Questionnaires completed (n) | 87 | 89 | 87 | 86 | 85 | 83 | 78 | 81 | 79 | 77 | 75 | 70 | 71 | 66 |
|  | Overall compliance (%) | 98 | 100 | 98 | 97 | 96 | 93 | 88 | 91 | 89 | 87 | 84 | 79 | 80 | 74 |
|  | Compliance of patients. alive (%) | 98 | 100 | 98 | 97 | 96 | 93 | 91 | 92 | 93 | 94 | 93 | 91 | 90 | 91 |
| Performance tests completed | |  |  |  |  |  |  |  |  |  |  |  |  |  |  |
|  | SPPB, (n) | 89 | 88 | - | - | - | - | 62 | 65 | 56 | 57 |  |  |  |  |
|  | Grip strength, (n) | 89 | 88 |  |  |  |  | 62 | 65 | 56 | 56 |  |  |  |  |
|  | SPPB, % of patients alive (% of overall group) |  |  |  |  |  |  | 72 (70) | 74 (73) | 66 (63) | 70 (64) |  |  |  |  |
|  | Grip strength % of patients alive (% of overall group) |  |  |  |  |  |  | 72 (70) | 74 (73) | 66 (63) | 68 (63) |  |  |  |  |
