# Supplementary material for: Geriatric assessment with management for older patients with cancer receiving radiotherapy: a cluster-randomised controlled pilot study
Source: BMC Med. 2024 Jun 10;22:232. doi: 10.1186/s12916-024-03446-4 (PMC11163782; doi:10.1186/s12916-024-03446-4)
Supplement: Supplementary file 5 — Additional file 5: Table S5. Initial GA results, prevalence of problems/needs in line with pre-defined guidelines and corresponding measures registered as implemented in log-notes. [file 12916_2024_3446_MOESM5_ESM.docx]

Additional file 5

**Additional file 5: Table S5.** Initial GA results, prevalence of problems/needs in line with pre-defined guidelines and corresponding measures registered as implemented in log-notes

|  | **Problem according to pre-defined guideline** | **Patients with registered problem**  **N (%)** | **Patients with registered problem and any measure implemented, N (% of patients with problems)** | **Type of measure implemented**  **(more than one for each patients possible)** |
| --- | --- | --- | --- | --- |
| **Somatic health** | | | | |
| **Extra lab tests** | |  |  | Notifications of oncologist, orally and/or in writing (5 registrations)  Referral to General Practitioner (GP) (15 registrations)  Previously recognised problem/condition (7 registrations) |
|  | Elevated HbA1c (glycated haemoglobin) | 25 (28) | - |  |
|  | Free thyroxine 4 (FT4) outside reference values | 6 (7) | - |  |
|  | Thyroid stimulating hormone (TSH) outside reference values | 11 (12) | - |  |
|  | Vitamin B12 outside reference values | 9 (10) | - |  |
|  | **Any of the extra test outside reference values** | **38 (43)** | **20 (53)** |  |
| **Blood pressure** | |  |  |  |
|  | **Mild to moderate hypertension** | **48 (54)** | **15 (31)** | Checked to confirm that control was scheduled (7 registrations)  GP control recommended (10 registrations) |
|  | Severe hypertension^a^ | 7 (8) | 1 (14) | 24 hours blood pressure registration recommended |
|  | Low blood pressure (< 100-110 systolic) | 2 (2) | 1 (50) | Checked for orthostatism |
| **Symptoms registered by ESAS scores (scored 0-10)** | |  |  | Notified oncologist (5 recordings)  Notified GP (11 recordings)  Symptom alleviating measures recommended (24 recordings) |
|  | Pain score > 4 | 12 (13) |  |  |
|  | Tiredness > 4 | 12 (13) |  |  |
|  | Drowsiness > 4 | 8 (9) |  |  |
|  | Nausea > 4 | 1 (1) |  |  |
|  | Lack of appetite > 4 | 6 (7) |  |  |
|  | Shortness of breath > 4 | 4 (5) |  |  |
|  | Depression > 4 | 3 (4) |  |  |
|  | Anxiety > 4 | 4 (5) |  |  |
|  | **Any symptom > 4** | **29 (33)** | **28 (97)** |  |
| **Comorbidities** | |  |  |  |
|  | **Impaired hearing** | **36 (40)** | **30 (83)** | Confirmed available hearing aids (26 registrations)  Need for hearing aids evaluated (2 registrations)  Hearing aids recommended (2 registrations)  Referral to hearing centre (1 registration) |
|  | Impaired vision | 30 (34) | 25 (83) | Confirmed available seeing aids (26 registrations) |
| Other comorbidities^b^ | |  |  |  |
|  | > 3 comorbidities according to Charlson Comorbidity index and/or > 3 comorbidities according to the Old American Resource Survey (OARS) (hearing and vision loss excluded) | 12 (14) | 4 (33) | Notification of GP (3 registrations) |
|  | Overall cohort | - | 7 (8%) of the overall cohort registered as receiving any measure related to comorbidity) | Notification of GP (3 registrations)  Discussed with other specialist (oncologist excluded)  Referred to other specialist (oncologist excluded) |
| **Medication** | |  |  |  |
|  | **> 5 regular medications^c^** | **36 (40)** | 19 (53) | Discussion with geriatrician (11 registrations)  Need of medication review with notification of oncologist, GP, or geriatrician (8 registrations)  Up-dating/matching of medication lists (1 registration) Otherwise, simple notifications of treating physicians |
| **Nutrition** | |  |  |  |
|  | MNA-SF 8 – 11 points (risk of undernutrition) | 23 (26) | 19 (83) | Mapping food intake (3 registrations), nutritional advice including advice on energy enrichment (14 registrations), nutritional supplements (1 registration), not wanting any further measures undertaken (5 registrations)  (Weight and risk of undernutrition were conveyed to the municipal cancer contact nurse for all 23 patients) |
|  | MNA-SF < 8 points (undernourished) | 2 (2) | 2 (100) | Two receiving nutritional advice, one referred to nutritionist |
| **Mental health** | | | | |
| **Cognition** | |  |  |  |
|  | Mini-Cog score below 4 | 23 (26) | 20 (87) | Information from next-of-kin retrieved (17 registrations)  Notification of oncologist and/or GP (6 registrations)  Recommendation of supportive aids (1 registration)  Plan for re-assessment (10 registrations) |
| **Depression** | |  |  |  |
|  | Geriatric Depression Scale (GDS) -15 score 5-8 (mild depression) | 6 (7) | 6 (100) | Various recommendations, i.e., for day-care centre, local cancer support centre, involvement of volunteer, increased physical activity (6 registrations). Plan for re-assessment (5 registrations), |
|  | GDS-15 score > 8 (moderate to severe depression) | 0 | 0 | - |
| **Function** | | | | |
| **Daily life activities** | |  |  |  |
|  | Inability in any basic activity of daily living (ADL)  (Barthel index < 19) | 6 (7) | 6 (100) | Recommendations for home care nursing and or occupational therapist (2 cases)  Needs already taken care of (4 cases). To be noted is that 3/6 patients already had home care nursing, 1 patient had practical help at home, 1 had private help and 4 had safety alarm. |
|  | Inability in any instrumental activity of daily living (IADL)  (Lawton sum score < 8, i.e., any grade of impairment in any area) | 27 (30) | 22 (81) | Needs already taken care of (20 registrations)  Recommendations for help from home care services (2 registrations) |
| **Mobility** | |  |  |  |
|  | Short Physical Performance Battery, Timed Up and Go (TUG), grip strength. | NA  No cut off for implementation of measures. Instructions to provide all patients with an adapted training programme | Of all patients in the intervention group: Mobility measures registered as implemented for 71 (80%) | Training programme (64 registrations)  Referral to physiotherapist (7 registrations) |
|  | Patients with falls within the last six months or TUG > 14 | 19 (21) | 9 (47) | Checked reasons for falling (7 registrations)  Notification of oncologist and/or GP (2 registrations)  Referral to home care services (1 registration)  Evaluation of occupational therapist recommended (1 registration)  Other falls-preventing measures recommended (4 registrations) |
| **Social conditions** | | | | |
|  | No specific guidelines given | NA | Measures registered for 3 patients (recommendations for day care centre/personal assistant/volunteer support | |

^a^Registered sitting diastolic values between 79 and 115 and systolic values between 161 - 207.

^b^There was no systematic registration of the patients fulfilling the guidelines for implementation of measures related to comorbidity (« Comorbidity affecting ADL or IADL, >3 comorbid conditions receiving active treatment and according to clinical judgment »), thus, to illustrate the amount of potential problems, the number of patients with > 3 comorbidities are displayed.

^c^There was no systematic registration of patients fulfilling the guidelines for implementation of measures related to medications i.e., “Use of a) medications with interactions classified as “should be avoided”, b) «STOP» medications or > 7 regular medication OR c) 5 regular medications + comorbidity affecting ADL/IADL or symptoms requiring new medication”, thus, to illustrate potential problems of polypharmacy, the number of patients with > 5 daily medications is cited.
